# Selected Mesoamerican Crops – Anti-Obesity Potential and Health Promotion. A Review

**DOI:** 10.1007/s11130-024-01211-9

**Published:** 2024-08-06

**Authors:** Talía Hernández-Pérez, Octavio Paredes-López

**Affiliations:** Centro de Investigación y de Estudios Avanzados del IPN (Instituto Politécnico Nacional), Irapuato, Guanajuato, 36824 México

**Keywords:** Nopal, Chia, Pumpkin, Cocoa, Health, Oxidative stress, Nutraceuticals

## Abstract

Mesoamerica is the center of origin of a great number of food crops that nowadays are part of a healthy diet. Pre-Columbian civilizations utilized more than 90% of these foods as ingredient or in main dishes, as well as for remedies and religious ceremonies. Since several years ago, Mesoamerican foods have been recognized by their outstanding concentration of bioactive compounds, including, phenolic compounds, pigments, essential fatty acids, amino acids, peptides, carbohydrates and vitamins, which provide a great number of health benefits. As a result of their unique composition, these ancient crops have several positive effects, such as hypoglycemic, antioxidant, anti-obesity, anti-inflammatory, anti-ageing, neuroprotective, anti-diarrheal, and anti-hypercholesterolemic capacity. Hence, this review is focused mainly in the anti-obesity and antioxidant potential of some of the most cultivated, harvested, as well as commercialized and consumed, food crops native of Mesoamerica, like, nopal and its fruit (*Opuntia ficus indica* spp.), chia (*Salvia hispanica* L.), pumpkin (*Cucurbita* spp.) and cacao (*Theobroma cacao*).

## Introduction

Mesoamerica is a historical region in America, which extends from the center of Mexico, through Belize, Guatemala, El Salvador, Honduras, Nicaragua and to the north of Costa Rica; it includes the whole area of Maya culture. It has been an important center of genetic diversity and is well recognized by its role in the plant domestication of different types of crops with outstanding characteristics [1]. One important heritage of Mesoamerican civilizations is the wide variety of plants for food and medicinal purposes. Nopal, chia, pumpkin and cacao are main crops of the modern diet, among others, which are important for people’s food security, providing nutritional and nutraceutical purposes. They have great potential to prevent or ameliorate non-communicable diseases, like obesity, dyslipidemia, and type 2 diabetes (Table 1) [2].

**Table 1:**
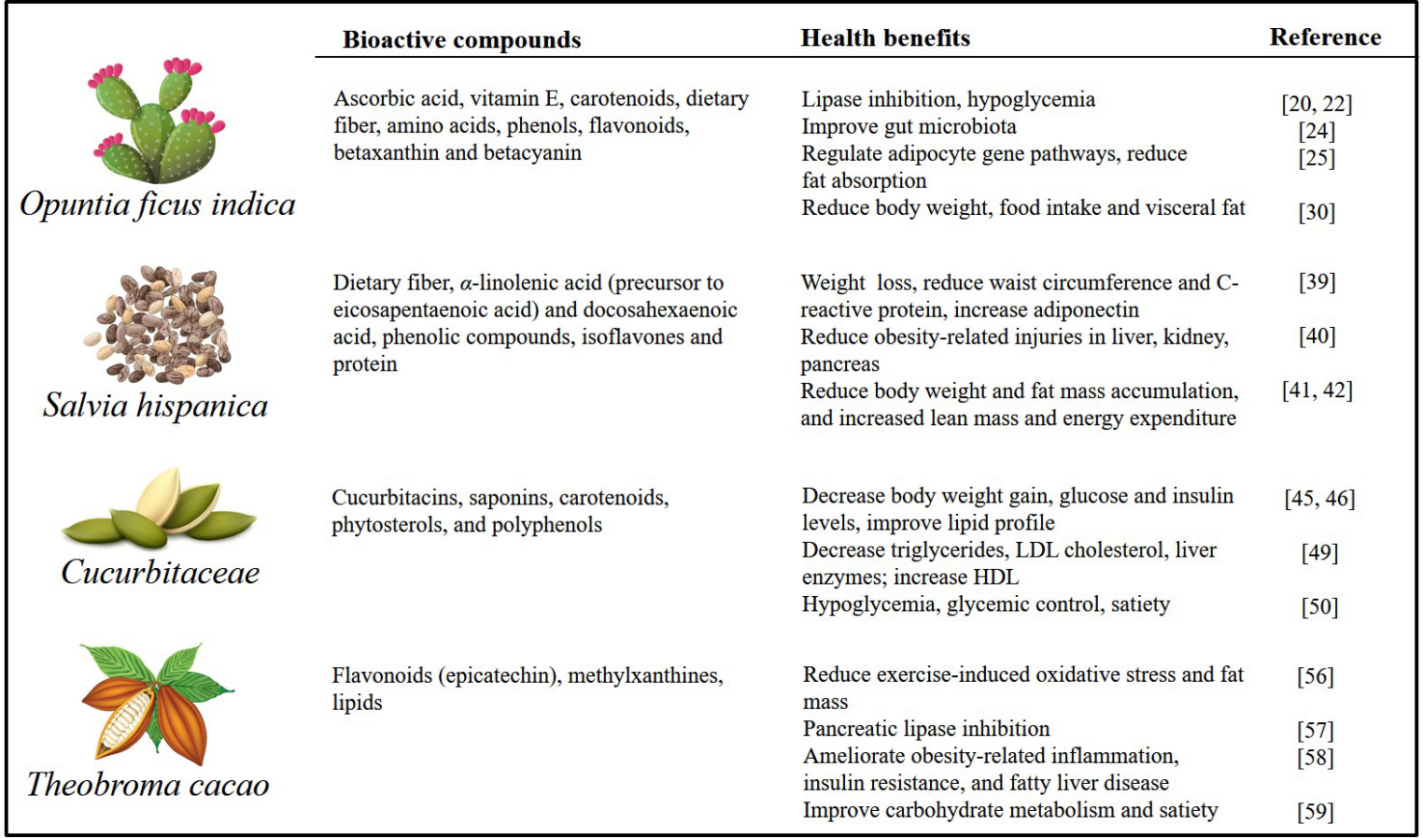
Anti-obesity potential of some Mesoamerican crops

Obesity is a multifactorial disease characterized by an excessive accumulation of fat in adipose tissue [3]. It is associated with insulin resistance, changes in the gut microbiota, resulting in disruption to the bacterial community (dysbiosis), affecting the host–microorganism relationship, energy homeostasis, metabolic function, and inducing inflammation [4, 5]. It is estimated that there are around 2 billion adults, over 18 years of age, currently living with overweight; and among them, 650 million are affected by obesity [body mass index (BMI) ≥ 30] which represents one of the world’s major public health problems [6, 7]; unfortunately, this trend keeps increasing. Visaria and Setoguchi [8] found that the risk of all-cause mortality is remarkably elevated among people with BMI ≥ 30. BMI may not necessarily increase mortality independently of other risk factors in adults, especially older adults, with overweight. Further studies incorporating weight history, body composition, and morbidity outcomes should be required for a full characterization of BMI-mortality associations. It could be a key strategy to include new plant ingredients of Mesoamerican origin into the traditional cuisine to promote a balanced diet and thus support weight management and prevent obesity [9].

## Anti-Obesity Potential of some Mesoamerican Food Plants

Lifestyle and environmental factors play a crucial role in the development of obesity [10]. Overweight and obesity promote the early onset of cardiovascular disease, diabetes, Alzheimer’s disease, and cancer, which are costly public health problems and leading causes of mortality worldwide. Many people solve this problem by using food supplements or drugs; they may contain food plant-derived molecules (primary and secondary metabolites, vitamins, and fibers), probiotics, and microbial-derived fractions (postbiotics). They may control lipid and carbohydrate metabolism, reduce appetite (interacting with the central nervous system) and adipogenesis, improve intestinal microbiota activity, and increase energy expenditure [11]. It is worth to mention that these food crops comprise important levels of proteins, which are the source of peptides with various biological roles. Their function is as signaling entities via all domains of life, and thus interfere with protein-protein interactions, which are indispensable in bioprocesses. In this context, short peptides show basic molecular information for health processes. They have attracted considerable attention due to their unique characteristics and outstanding potential for novel biotherapies [12]. As result of their high concentration of nutraceutical compounds, the intake of nopal, chia, pumpkin and cacao are a safe alternative for the prevention and treatment of obesity and promotion of human health.

## *Opuntia ficus-indica*

### Nopal –Pad and Fruit

**Fig. 1:**
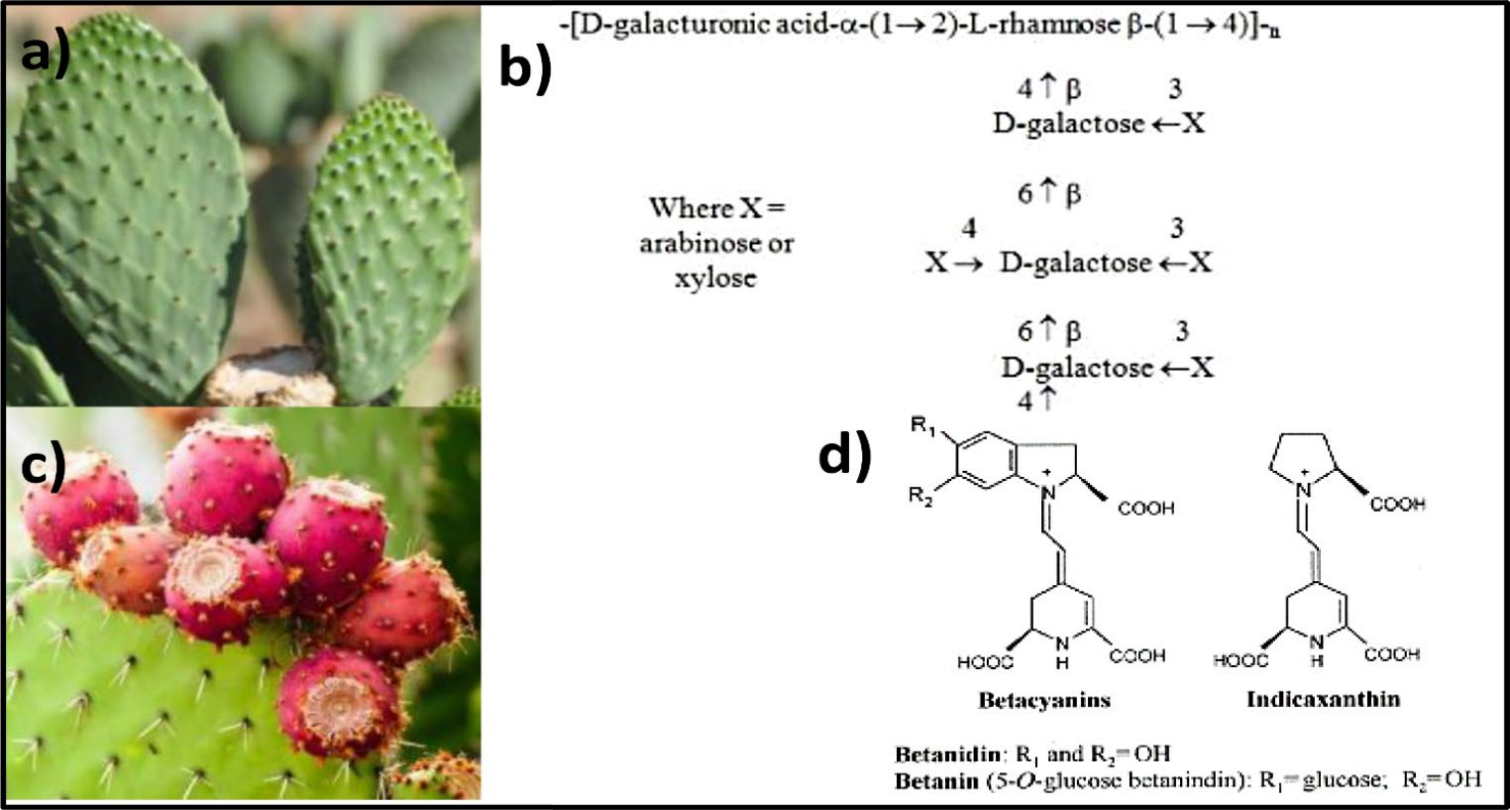
**a. ***Opuntia ficus* cladodes, **b.** Chemical structure of mucilage, **c.** Prickly pear fruit, **d.** Betalains, most abundant pigments in prickly pear [21, 27]

*Opuntia* spp. belongs to the Cactaceae family, which includes more than 300 species and most of them are native to Mesoamerica (Fig. 1a). *Opuntia ficus-indica* L. Mill. is the most widely consumed species of the *Opuntia* genus due to its tasty cladodes and fruits. Nopal (*O*. *ficus-indica*), commonly known as prickly pear cacti, is widely distributed in Mexico, other Latin American countries, the Mediterranean area and South Africa. In view of its performance under difficult environmental conditions, it has been classified as “one of the most prominent food crops for the 21^st^ century”. Besides, *Opuntia* cladodes and fruits are valuable crops due to their attractive horticultural characteristics, functional compounds, and ease of cultivation conditions [13, 14]. Its cladodes have been used, besides their common consumption in different food dishes, in traditional medicine as an anti-ulcerogenic, antidiarrheal, antioxidant, anti-inflammatory, hypoglycemic, neuroprotective, and anti-hypercholesterolemic agent. These properties are attributed to the presence of ascorbic acid, vitamin E, carotenoids, dietary fiber, amino acids, and antioxidant compounds (phenols, flavonoids, betaxanthin and betacyanin) [15–17].

Since the past decades, a great interest has emerged on the role of this crop in the treatment of metabolic syndrome, which is mostly associated to type 2 diabetes [18, 19]. Kalegowda et al. [20] found that mucilage from *Opuntia dillenii* (Ker-Gawl) cladodes exhibited anti-obesity properties through lipase inhibition. Therefore, the isolated mucilage could be an ingredient of food and pharmaceuticals (Fig. 1b) [21]. In addition to glycemic control, *Opuntia* cladodes may counteract the negative metabolic effects of the Western diet [22].

Sánchez-Tapia et al. [23] observed, in a murine model, that a nopal diet intervention modified the gut microbiota and reduced the metabolic consequences of obesity, protecting from metabolic endotoxemia, and increasing the level of the protein occludin1 in the colon, thus reducing gut permeability. In this sense, Corona-Cervantes et al. [24] found that caloric restriction, physical activity plus the addition of nopal into the diet could modify the gut microbiota in obese women. It also improved the host metabolism, as suggested by the correlation between some bacterial species with biochemical and anthropometrical parameters. Besides, Héliès-Toussaint et al. [25] observed that powder from *Opuntia*’s cladodes reduces intestinal fat absorption by regulating adipocyte differentiation genes pathway, and improves gut microbiota. Hence, *O. ficus-indica* is a promising nutraceutical to manage obesity and its metabolic implications, such as hepatic diseases.

Prickly pear fruits are available in different colors (Fig. 1c); mainly white, red, green, yellow, purple, and orange colored pulps, which have been characterized in the last years and are rich in several bioctive compounds. Valero-Galván et al. [26] found that wild prickly pear fruits comprise phenolic compounds, flavonoids, antioxidants, protein, lipids, ascorbic acid, fiber, and betalains composed by betacyanins (red-violet), and betaxanthins (yellow-orange) (Fig. 1d), with outstanding antioxidant properties and thus biological interest, and can be a potential source of functional ingredients and nutraceuticals [27]. Current dietary recommendations for cardiovascular disease risk reduction include increased fruit and vegetable consumption. Prickly pear fruit is rich in dietary fiber and may have lipid-lowering effects. Gouws et al. [28] found that in healthy and obese populations as well as those with metabolic illnesses, specifically type 2 diabetes and metabolic syndrome, *Opuntia* fruits reduced total cholesterol and low-density lipoprotein cholesterol. Isorhamnetin and its derivatives, flavonoids found in some *Opuntia* species, showed a positive effect on adipogenesis and triglyceride accumulation in cultured adipocytes [29].

The negative metabolic effects of obesity are the most common causes of death globally. Indicaxanthin, a betalain pigment found in the fruit of *O. ficus indica*, modulates redox dependent signaling pathways, thus exerting significant antioxidant and anti-inflammatory effects in vitro and in vivo. In a high-fat diet (HFD) model of obesity-related insulin resistance, indicaxanthin significantly reduced body weight, daily food intake and visceral fat mass. It also showed benefits on HFD-induced glucose dysmetabolism, reducing fasting glycaemia and insulinemia, improving glucose and insulin tolerance and restoring the HOMA index to physiological values. These effects were associated with a reduction in hepatic and adipose tissue oxidative stress and inflammation. Additionally, there was a decrease in reactive oxygen and nitrogen species and inflammatory gene expression [30].

Enriched bread with roasted prickly pear seed flour, at different levels, showed total phenolics, flavonoids and radical scavenging activity several folds higher when compared to wheat flour. It also increased dietary fibers, fat, and ash contents and reduced the carbohydrate content of the produced breads [31].

### Chia

**Fig. 2:**
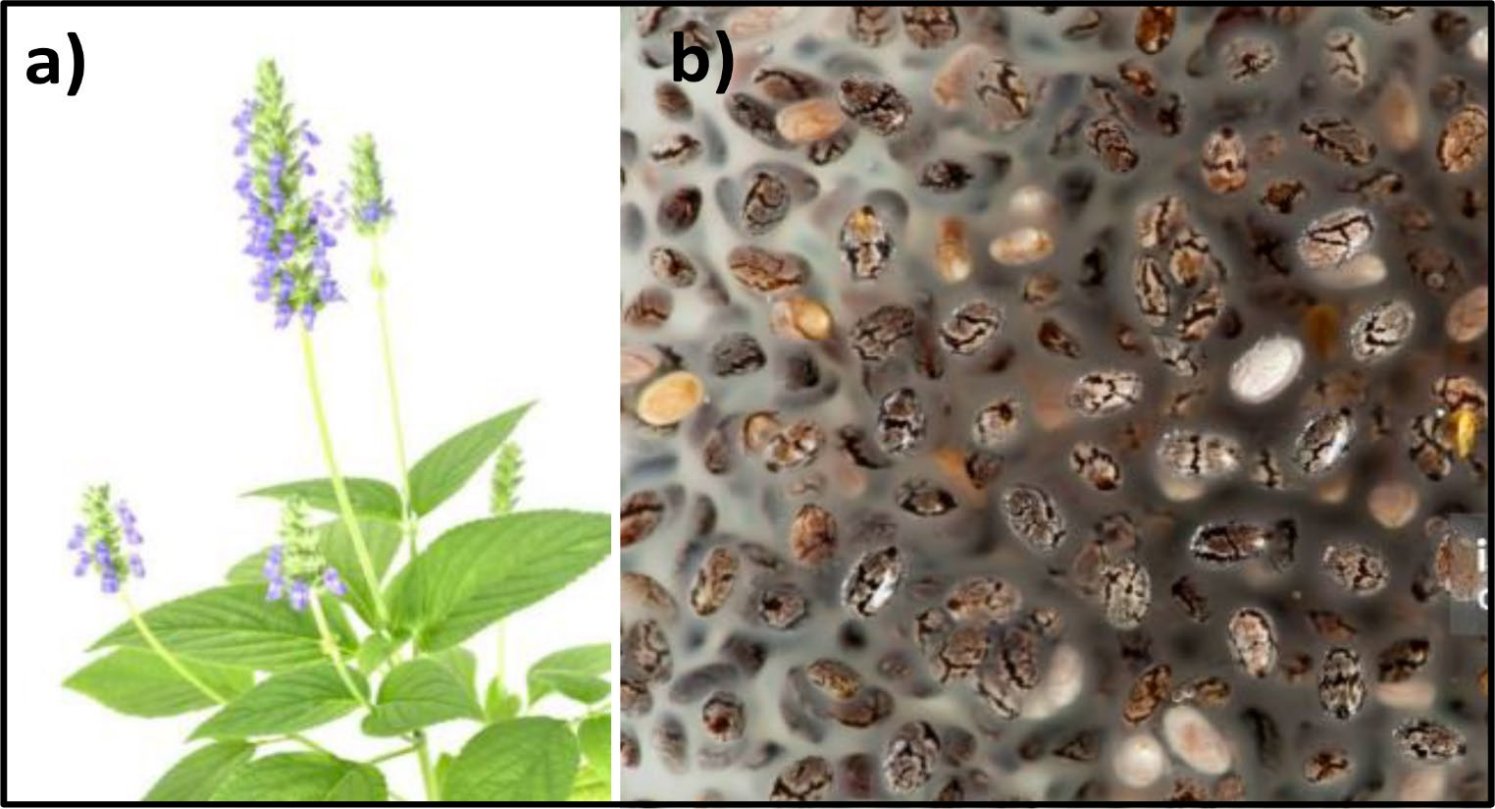
**a. ***Salvia hispanica* plant, **b.** Chia mucilage contanins 72% soluble fiber [34, 35]

In the last decades, chia (*Salvia hispanica*) has been rediscovered and recognized as an ancient crop with remarkable potential benefits for human health. This is primarily due to its high concentration of ω-3 and ω -6 fatty acids, soluble mucilage (Fig. 2a) and insoluble fibers, as well as proteins with an important concentration of essential amino acids, minerals, vitamins, and phytochemicals with high antioxidant activities including phenolics and isoflavones. They have been isolated and evaluated to understand their functional properties, and nutritional and health benefits [32, 33]. Additionally, chia mucilage contains ~ 72% soluble fiber (Fig. 2b) in addition to monosaccharides, mainly arabinose and xylose, followed by glucose, fructose, galactose, rhamnose, and mannose [34, 35].

Nowadays, *S. hispanica* seeds are used to prevent non-infectious diseases, *i.e*., obesity, hypertension, cardiovascular diseases, cancer and diabetes [36]. Pelaez et al. [37] using transcriptomic analysis have reported that chia is native to the occidental region of Mexico and to Guatemala. Chia, wild and cultivated, was a very important plant before the arrival of the Spanish conquerors; it is well known that the Aztec emperor, Moctezuma, and his court and soldiers were frequent consumers of chia seeds. During periods of hard work or before battles, beverages based on this seed were widely consumed because of its lipids and fatty acids gave them energy, and periods for drinking water were not so common since the seed oil gave some protection to the stomach walls. The cultivation of this crop was forbidden by the new authorities, for religious matters and thus, chia became a forgotten crop.

Chia seeds exerted significant effects on adiposity related parameters when given with an obesogenic diet in rats [38]. After a 6-month calorie-restricted diet, diabetic obese patients showed more weight loss than control, accompanied by a higher reduction in waist circumference. C-reactive protein was also reduced in chia group, compared to control, and plasma adiponectin increased by 6.5 ± 0.7%; these seeds may be a useful dietary supplement to treat obesity and diabetes [39].

The modification of the daily diet is the primary tool to control or prevent obesity. Sprague-Dawley rats fed a high-fat diet supplemented with the combination of potential prebiotics (chia seeds, green tea, and chitosan) for 45 days, showed a significant decrease in body weight. They also decreased serum catalase, superoxide dismutase, and liver malondialdehyde levels, as well as obesity-related injuries in the liver, kidney, and pancreas. Further docking studies indicated the potential role of these seeds and green tea components in modulating obesity [40]. Fonte-Faria et al. [41] observed that obese mice fed with a high-fat diet and chia oil from 90 to 135 days, reduced fat mass accumulation and increased lean mass as evidenced by nuclear magnetic resonance. They also improved glucose levels and insulin tolerance, decreased serum leptin and triacylglycerols, and increased LDL (low density lipoproteins) cholesterol.

Quaresma et al. [42] evaluated the influence of chia flour intake (90 days) on body weight, body composition, energy expenditure and cardiovascular risk in obese women. Chia showed positive effects on systolic blood pressure, but negatively affected lipid profile (decrease in HDL, high density lipoproteins) and did not influence obesity control.

### Pumpkin Seeds

**Fig. 3:**
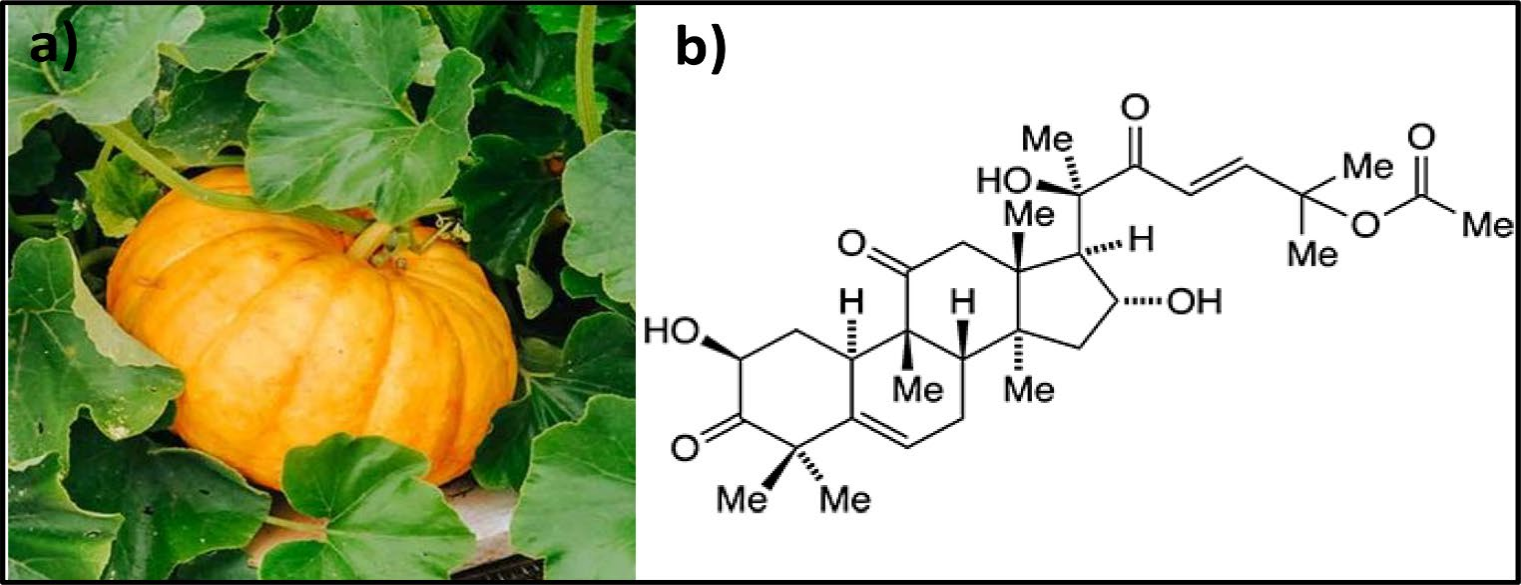
**a. ***Cucurbita* sp. plant, **b.** Cucurbitacins, highly potent pharmacological agent [45, 48]

Pumpkins are native to Mesoamerica. The New World was the center of cucurbit domestication in pre-Columbian times, around 11,000 years ago. Some species of the *Cucurbita* genus, *C. pepo*, *C. maxima*, *C. argyrosperma* and *C. moschata*, are of worldwide economic relevance and cultivated globally [43]. Plants from *Cucurbita* speces are rich sources of phytochemicals (Fig. 3a).

*Cucurbita* species comprise high levels of carotenoids, cucurbitacins, saponins, phytosterols, polyphenols, and fiber, which show antioxidant, anti-obesity, antitumor, antidiabetic, hepatoprotective, antimicrobial, diuretic, anti-ulcer, and antigenotoxic capacity. Because of these characteristics, pumpkin is considered as a super food. Clinical trials also suggest that *Cucurbita* is useful for benign prostate hyperplasia, as well as infertility, postmenopause and stress urinary incontinence in women [44]. Oral administration of *Cucurbita maxima* seeds oil (CSO) (100 mg/kg body weight; 30 days) in HFD-induced obese rats revealed significant diminution in body weight gain, glucose and insulin levels, which altered the activity of lipid profile and restored the pathological alterations. CSO might ameliorate HFD-induced obesity by altering the enzymes and mRNA expressions important to lipid metabolism [45, 46]. Cucurbitacins are found in many cucurbitaceous plants; it is noteworthy that cucurbitacin exerts an anticancer effect by inhibiting telomerase, among other underlying mechanisms (Fig. 3b). Although some cucurbitacins may be toxic compounds, most of them possess numerous pharmacological effectiveness against inflammation, cancer, atherosclerosis and diabetes. The reports on their toxicity must not overshadow the potential use of these compounds as important medicinal agents [47, 48].

Obese male Wistar rats receiving pumpkin seed extracts (100, 200, or 400 mg/kg) once daily for six weeks, dramatically decreased triglycerides, LDL cholesterol and liver enzymes, while HDL was markedly increased. Hence, the capacity of pumpkin to ameliorate oxidative stress and dyslipidemia leads to reductions of heart conditions in obese patients [49]. Adams et al. [50] demonstrated that trigonelline, nicotinic acid, and d-chiro-inositol from pumpkin seeds possess hypoglycemic properties and could assist in maintaining glycemic control and satiety.

### Cacao

**Fig. 4:**
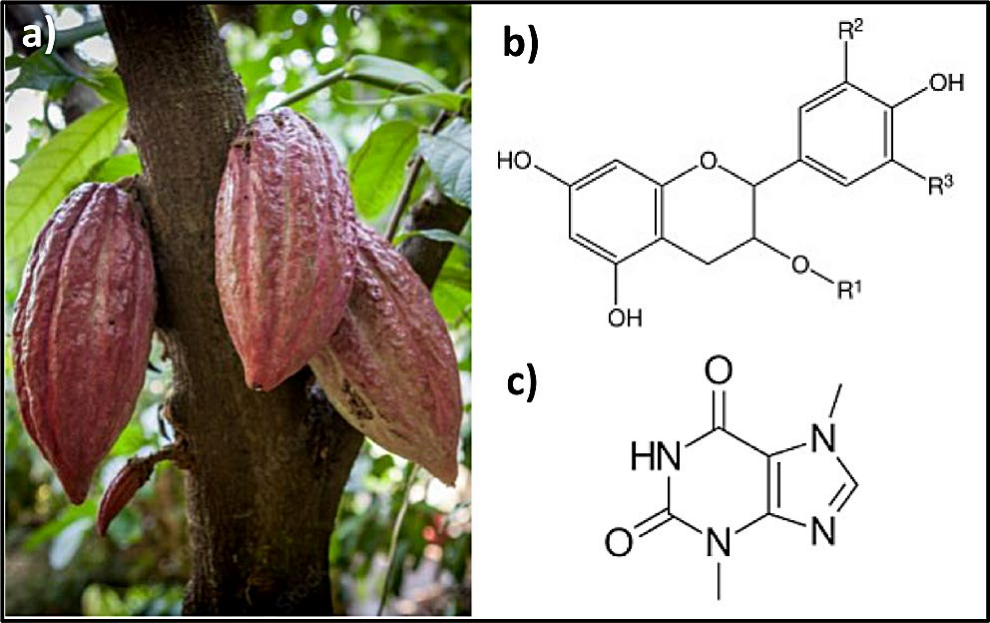
**a. ***Theobroma cacao* fruits, **b.** Flavanols, **c.** Thebromine, main bioactive compounds of cacao [56, 57]

*Theobroma cacao*, “The Food of the Gods”, provides the raw material for the multibillion-dollar chocolate industry, besides, it is also used as ingredient for the pharmaceutical and cosmetic industries. Cocoa powder is a product obtained from the beans of the *T. cacao* plant, which is a rich source of fiber (26–40%), proteins (15–20%), carbohydrates (around 15%) and lipids (10–24%) (Fig. 4a) [51]. It also provides minerals and vitamins. It is rich in polyphenols, particularly flavonoids (epicatechin), methylxanthines, and other compounds, which have positive effects in cardiovascular and neurodegenerative diseases, diabetes and weight reduction [52, 53]. The use of cacao in health dates back at least 3,000 years. The high concentration of epicatechin in dark chocolate controls blood pressure, lipids, and inflammation due to enhanced nitric oxide bioavailability and improved mitochondrial structure/function [54, 55].

Decroix et al. [56] suggested that cocoa flavonoids improve exercise performance and recovery, vascular function, reduce exercise-induced oxidative stress, and alter fat and carbohydrate utilization during exercise. Additionally, Coronado-Cáceres et al. [57] observed that cocoa peptides inhibited pancreatic lipase (PL) (IC_50_ < 1.38 mg/mL) and intragastric administration of 150 mg cocoa protein/kg/day to rats increased total lipids and triglycerides excretion in feces. In addition, HFD + cocoa protein-diet significantly decreased fat absorption compared with the high fat group. Therefore, cocoa has anti-obesity potential by inhibiting PL and thus prevents the development of non-communicable diseases.

Gu et al. [58] found that 8% unsweetened cocoa powder (approximately equivalent to 465 mg/ kg/d in humans) for 10-weeks significantly ameliorated obesity-related inflammation, insulin resistance, and fatty liver disease in high-fat mice. Dark chocolate, a high source of polyphenols (flavanols), modulates obesity due to its potential effect on fat and carbohydrate metabolism, as well as on satiety (Fig. 4b) [59]. It is noteworthy that cocoa powder has become a subject of increasing interest due to its high content of flavonoids, mainly constituted by procyanidins, compounds with antioxidant activity as well.

Cocoa polyphenols are not exclusively responsible for gut microflora alterations; other phytochemicals, such as theobromine, influence and modify gut microbiota (Fig. 4c). As it has been examined in rodents, a cocoa containing diet which is rich in theobromine and fiber has more modulatory effects on gut microbiota than polyphenols [60].

In brief, clinical research suggests that cocoa and its flavonoids may play a key role for the nutritional management of cardiovascular diseases, obesity and diabetes, among other chronic diseases. Cocoa polyphenols and their secondary metabolites can modulate the microbiota composition and function; these compounds together with the high percentage of dietary fiber present in cocoa, as indicated above, may well explain the prebiotic functionalities that have been attributed to cocoa such as gut microbiota modifications with positive impact in human health [60].

## Conclusions

Metabolic diseases such as obesity, cardiovascular diseases, and type 2 diabetes are exacerbated by the modern food system that provides mainly ultra-processed high-fat and high-sugar diets complemented by a sedentary lifestyle, thus exposing the population across all age groups to an increase of these diseases. All of them constitute the metabolic syndrome, which represents nowadays a major public concern at the worldwide level. Dietary factors are important in preventing the onset of these metabolic conditions; consuming foods that provide health benefits, in addition to nutrition, is essential for the prevention of such diseases. The inclusion of plant foods from Mesoamerican origin, like those from the selected crops described here, has demonstrated their capacity for helping in the treatment of overweight, obesity and in the promotion of other health benefits owing to their richness in bioactive compounds. In a brief way, this review presents mainly the findings from recent and relatively recent studies on the health potentialities of the components provided by these plant foods from the Mesoamerican subcontinent; further studies are needed, based on the up-to-date knowledge, for the improvement of the future nutrition, nutraceutical and medicinal key role during the 21^st^ century of these crops, and their extracted compounds.

## Data Availability

No datasets were generated or analysed during the current study.
